# Lateral gene transfer of an ABC transporter complex between major constituents of the human gut microbiome

**DOI:** 10.1186/1471-2180-12-248

**Published:** 2012-11-01

**Authors:** Conor J Meehan, Robert G Beiko

**Affiliations:** 1 Faculty of Biochemistry and Molecular Biology, Dalhousie University, 5080 College Street, Halifax, NS, B3H 4R2, Canada; 2 Faculty of Computer Science, 6050 University Avenue, Halifax, NS, B3H 1W5, Canada

## Abstract

**Background:**

Several links have been established between the human gut microbiome and conditions such as obesity and inflammatory bowel syndrome. This highlights the importance of understanding what properties of the gut microbiome can affect the health of the human host. Studies have been undertaken to determine the species composition of this microbiome and infer functional profiles associated with such host properties. However, lateral gene transfer (LGT) between community members may result in misleading taxonomic attributions for the recipient organisms, thus making species-function links difficult to establish.

**Results:**

We identified a peptides/nickel transport complex whose components differed in abundance based upon levels of host obesity, and assigned the encoded proteins to members of the microbial community. Each protein was assigned to several distinct taxonomic groups, with moderate levels of agreement observed among different proteins in the complex. Phylogenetic trees of these proteins produced clusters that differed greatly from taxonomic attributions and indicated that habitat-directed LGT of this complex is likely to have occurred, though not always between the same partners.

**Conclusions:**

These findings demonstrate that certain membrane transport systems may be an important factor within an obese-associated gut microbiome and that such complexes may be acquired several times by different strains of the same species. Additionally, an example of individual proteins from different organisms being transferred into one operon was observed, potentially demonstrating a functional complex despite the donors of the subunits being taxonomically disparate. Our results also highlight the potential impact of habitat-directed LGT on the resident microbiota.

## Background

A vast array of bacteria, archaea, viruses and eukaryotes inhabit the tract of the human gut and form its microbiome [1,2]. Investigation into the composition of this densely packed community and its effect on the host have revealed several benefits derived from the microorganisms such as plant polysaccharide processing and amino acid synthesis [1,3]. The species structure of the community has also been linked to several health problems such as inflammatory bowel disease [4] and obesity [5-7].

Initial studies of the human gut microbiome involved sequencing of the 16S ribosomal RNA gene to determine the main constituents of the community. Although many organisms observed in these studies were previously uncharacterised [8], members of the phyla Firmicutes and Bacteroidetes comprised over 90% of the population of known bacterial species within the gut [4]. The Human Microbiome Project (HMP) utilised both a 16S-based approach and a large-scale study of obese and lean twin pairs, and found that the species composition of the gut microbiome was more similar in related individuals than unrelated individuals [7]. However no core species group was observed in all studied individuals. A preliminary investigation of full genome sequences was also performed on a subset of samples in this study, revealing that similar taxonomic profiles were linked to similar metabolic profiles between individuals [7]. Each of the two main phyla (Firmicutes and Bacteroidetes) was associated with enrichment of different metabolic pathways (transporters and carbohydrate metabolism respectively) and although the species composition differed between individuals, there was a relatively high level of functional conservation in the majority of gut microbiomes studied.

Associative studies between the human gut microbiome and host factors such as inflammatory bowel disease (IBD) and weight have revealed close ties between the composition of the microorganism community and human health [4,6,9,10]. Metagenomic sequencing of faecal samples from 124 European individuals was performed in order to study multiple portions of the community gene pool and link variation in community to IBD [4]. A core gut microbiome gene pool was reported along with a proposed list of possible core species. These species were primarily from the two main phyla identified previously, and taxonomic rank abundances were used to distinguish between IBD and non-IBD individuals. Taxonomic differences have also been linked to obesity, especially based upon relative abundances of different phyla. Turnbaugh et al. found that obese twins had a lower proportion of Bacteroidetes than lean twins [7]. This relationship between weight and the reduction of Bacteroidetes species has also been supported by other studies [5,10]. However, additional studies have found either no significant change in the Firmicutes: Bacteroidetes ratio [6,11] or even an increase in Bacteroidetes in obese individuals [12].

The aim of our study was to investigate whether links could be made between an individual’s body mass index (BMI) and metabolic functions within the microbiome. Findings indicate that multiple components of the peptides/nickel transport system show consistent differences in abundance based upon levels of obesity within the sampled individuals. This transporter is comprised of five proteins and is likely used to transport nickel into cells and regulate its intracellular concentration [13], or potentially regulate the expression of cell surface molecules through selective uptake of short peptides [14]. Despite significant differences in the abundance of complex members, the taxonomic distribution of these proteins did not differ between obese and lean individuals. However, phylogenetic analysis of abundant species, regardless of BMI, revealed that these proteins were likely laterally acquired from other gut-associated microbes, indicating that habitat-directed LGT can influence microbial metabolic systems that are linked to human health.

## Results and discussion

### Dataset processing

Prediction of open reading frames (ORFs) from the dataset of 124 patients presented in [4] revealed an average of 203,300 potential ORFs per sample. Use of BLAST sequence matching resulted in predicted protein functions for, on average, 46% of the ORFs per sample. Subsequent characterisation of these putative protein sequence fragments using the KEGG database allowed for metabolic classification of 39% of the ORFs with BLAST hits (18% of the original predicted ORF set). Each microbiome sample had an average of 2,400 KO groupings containing at least one sequence fragment with a total of 4,849 KOs being present in at least one sample in the dataset.

### Distributions of predicted metabolic functions between low and high-BMI groups

**Figure 1:**
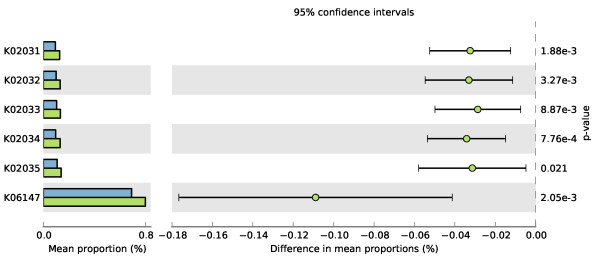
**KOs that differ significantly between lean (green) and obese (blue) individuals.** Statistical analysis of all KOs within a patient revealed five that differ in proportions with mean abundance greater than 0.2%. Mean abundance within a group (green = lean, blue = obese) are demonstrated by the bar charts (relative to the total number of ORFs assigned to KOs in the dataset; total number of sequenced assigned is 1,389,124) and the percentage differences between groups are shown on the right with the green circle indicating that a higher proportion is present in lean individuals.

Sequence counts for all 4,849 KOs were compared across patients in order to identify metabolic functions that differ in abundance between low BMI (18 to 22) and high BMI (30+) associated samples. Present KEGG Orthology groups ranged in relative abundance from 4 × 10^-5^ (i.e. one copy of the protein in the largest sample) to 0.8% of the total assigned proteins, with K06147 (bacterial ATP-binding cassette, subfamily B) as the most abundant KO across all patients, regardless of BMI. Fifty-two KOs were found to differ significantly (Bonferroni-corrected p value <0.01) in abundance levels between lean- and obese-related samples. The majority of these KOs were low in frequency in both BMI categories; apart from the ABC transporter mentioned above, only five of the 52 KOs had a mean proportion in both BMI sets of 0.2% or higher (Figure 1). K06147, in addition to being the most abundant protein in all patients, was 46% more abundant in low-BMI samples. The other four KOs that were found to have significant differences in abundances all belong to the peptides/nickel transport system module (KEGG module M00239). This module contains five ABC transporter proteins (K02031-K02035), four of which were found to be significantly more abundant in low-BMI patients (K02031-K02034; ratios ranging between 42 and 44%; corrected p-values < 0.01) (Figure 1). This transport system contains two ATP-binding proteins (K02031 and K02032), two permeases (K02033 and K02034) and one substrate-binding protein (K02035). Variation in abundances of each KO between patients in the same BMI group (lean or obese) was found to be low, with mean proportions at most 0.2%. Although differences in abundance of K02035 were not found to be as statistically supported as the other subunits (p-value 0.021) it was found at similar levels of abundance between patients as the other four members of the transport system. Thus K02035 was included alongside the other subunits in the module in order to identify if specific species are associated with the complex as a whole.

### Taxonomic assignment of metagenomic fragments associated with nickel transporters

Reference phylogenetic trees were constructed for each of the five KOs within the peptides/nickel transport complex using proteins from 3,181 sequenced genomes retrieved from IMG [15] (Additional file 1: Figure S1). Habitat metadata from the IMG database [15] was used to assign species to the human gastrointestinal tract resulting in 472 gut-associated species. It was found that these species were spread throughout the trees and did not appear to cluster based upon habitat (Additional file 1: Figure S1). We constructed subtrees containing only gut-associated species and assessed the cohesion of taxonomic groups using the consistency index (CI): CIs close to 1.0 indicate perfect clustering of all taxonomic groups at a particular rank, while low CIs indicate intermingling of organisms from different groups and are suggestive of LGT, especially if organisms in the same cluster are from very disparate groups. The CIs of all trees were less than 0.5 when evaluated at the ranks of family, class, order and phylum (Additional file 2: Table S1), suggesting a lack of cohesion of major lineages. CIs at the genus (0.60 to 0.64) and species (0.93 to 0.96) levels were higher, indicating less disruption of these groups. Examples of disrupted species include *Faecalibacterium prausnitzii* and *Clostridium difficile* in the tree of K02031 sequences from gut-associated species (Additional file 3: Figure S2); in this case, large evolutionary distances separated sequences associated with strains of the same species. However as such disparities were also observed within the trees containing all species, not just gut-associated strains, further analysis was required to discover whether LGT events were directed by environment.

Pplacer [16] was used to place metagenomic fragments onto expanded reference trees for each of the KOs of interest. Not all fragments were mapped down to species level and thus a proportion was assigned only to a rank of genus or higher. The quantity of reads that were unclassified at different levels due either to lack of placement confidence of the read below a certain taxonomic level or lack of NCBI taxonomy information varied between KOs (Table 1). Taxonomic assignment was above 75% at all levels of classification with an average of 93% per rank. Fragments that were not mapped below a certain level were labelled as ‘unclassified’ and disregarded in further abundance analysis at that level. In general, Firmicutes were the dominant phylum associated with each KO, as is to be expected by their abundance within the gut [4], with the class Clostridia and order Clostridiales making up the largest proportion of classified reads in each sample. Several Firmicute genera, including *Clostridium*, *Blautia*, *Ruminococcus* and *Faecalibacterium*, were found to be in relatively high abundance in almost every protein set (up to 15%). Members of other phyla such as Proteobacteria and Actinobacteria also contributed to the species composition of proteins within this complex though these signals were less abundant and consistent than the Firmicute members. Thus, although correlation of assignments at higher taxonomic ranks was found between KOs, this did not extend to the genus level. This could be due to incorrect taxonomic assignments as a result of a deficiency in relevant reference genomes or lack of predictive power from the metagenomic ORFs. Inconsistencies could also be due to recent LGT events between members of different genera, which would result in discordant taxonomic assignments associated with the recipient species. Thus it is possible that this protein complex is present in a smaller, more consistent, set of genera with the human gut microbiome than is observed here.

**Table 1:** Percentage of reads assigned at each taxonomic level for each protein in the peptides/nickel transport system

**KO**	**Phylum**	**Class**	**Order**	**Family**	**Genus**	**Species**
**K02031**	98.11	96.61	96.36	91.1	84.71	75.56
**K02032**	99.68	99.45	99.26	98.06	96.2	93.52
**K02033**	98.61	97.9	97.3	93.28	83.68	77.91
**K02034**	99.64	99.54	99.32	97.9	95.61	90.28
**K02035**	98.21	94.93	94.62	86.84	84.35	77.13

Mapping of species classifications revealed further disparate signals between the KOs. Within each of the proteins K02031-K02035, no single species was represented in more than 9% of taxonomic attributions (Table 2). Collectively, the top four contributing species did not comprise more than 25% of the taxonomic groups associated with any of these KOs. As many of the fragments were not classified to the species level (average of 17.12%), it is difficult to determine exactly what species are most commonly associated with each protein. Analysis of the peptides/nickel transport system revealed very little overlap in species composition between the individual proteins of the complex. Only *Faecalibacterium prausnitzii* was found in relatively high abundance in all five KO phylogenies, with most other highly abundant species only being highly associated with at most three components. However, all of the most abundantly associated species are resident within either the gut or the oral cavity of the human microbiome. Thus, despite low overlap of species composition, fragments were found to be derived from microbes associated with the human alimentary canal as is to be expected.

**Table 2:** Percentage of four most abundant species associated with each protein

**Species**	**K02031**	**K02032**	**K02033**	**K02034**	**K02035**
*Blautia hansenii*	**3.4**	1.22	**3.99**	**3.63**	0.03
*Clostridium hathewayi*	1.31	**3.01**	0.98	1.49	0.26
*Clostridium phytofermentans*	3.04	**2.68**	2.6	**5.65**	0.02
*Clostridium proteoclasticum*	0	1.13	**3.65**	0.66	0.83
*Dialister invisus*	1.53	0.44	**3.15**	**2.83**	**4.02**
*Eubacterium rectale*	**3.44**	**2.13**	**2.39**	2.79	0.43
*Faecalibacterium prausnitzii*	**5.99**	**2.45**	**6.02**	**8.1**	**9.4**
*Oribacterium sinus*	0.31	**2.18**	0	0	0
*Roseburia inulinivorans*	**4.17**	0.97	1.99	**4.43**	**1.52**
*Salmonella enterica*	**0.69**	0.44	1.24	0.78	**6.15**
unclassified	**24.44**	**6.48**	**22.09**	**9.72**	**22.87**
*Xenorhabdus nematophila*	0	0	0	0	**4.5**

The most abundant species associated with each KO within the peptides/nickel transport system are shown here. The five most abundant species in each KO are highlighted in bold and also listed for every other KO.

### Analysis of *Faecalibacterium prausnitzii* strains within reference protein phylogenetic trees

**Figure 2:**
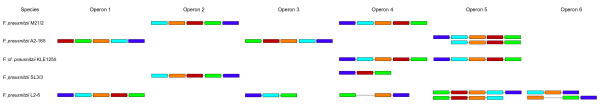
**Arrangement of peptides/nickel transporter operons within the five strains of *Faecalibacterium prausnitzii.*** Phylogenetic analysis of sequences associated with the nickel/peptides transporter complex revealed six distinct operons of potentially different origins. Operon constituents are coloured by KO (red = K02031; green = K02032; blue = K02033; orange = K02034; purple = K02035) with operon order according to numbering of genes in IMG chromosome maps.

The probable origin of each subunit of the peptides/nickel transport system within *F*. *prausnitzii* was examined using full-length protein trees derived from 3,181 sequenced species. It was found that the five sequenced strains of this species (M21/2, A2-165, KLE1255, SL3/3 and L2-6) contained up to 6 copies of each gene, which were spread across up to six operons with an average of 2.8 per strain (Figure 2). Operons were classified based upon whether the strains formed a closely related group within the full protein tree of the constituent KOs. Up to six such groups were found within each protein tree for K02031-K02035, resulting in the postulation of six operon types, each with a potential separate origin. Each operon type appeared to be derived from an LGT event from strains of various taxonomically spread species (Additional file 4: Figure S3). However, most of these species are associated with the human gut microbiome, suggesting that habitat-direct LGT occurred. Operon 3, which is complete only in strain A2-165, appears to have been potentially acquired from multiple bacterial species with the ATP-binding proteins (K02031 and K02032) separately acquired from the remaining proteins (Additional file 4: Figure S3). Gene neighbourhood analysis revealed preservation of operon organisation between *F*. *prausnitzii* strains and potential donors of operons, though not similarity in flanking regions, adding credence to the possibility of LGT resulting in acquisition of this function. Although multiple strains of *F*. *prausnitzii* contain each type of operon, suggesting acquisition prior to strain separation, rearrangement of the gene constituents appears to be frequent with inversions observed in operon types 2 and 5 and potential loss of components in operons 3, 4, 5 and 6 (although sequence similarity between missing sections of operon 5 in strains A2-165 and L2-6 and K02035 indicate this gene is present, though not annotated correctly).

Although high abundance of *F*. *prausnitzii* was found in association with the peptides/nickel transport complex, regardless of BMI, analysis of the species abundance associated with changes in BMI revealed no noticeable difference between low and high BMI patients. This could be due to the high numbers of unclassified reads, several cases of LGT confusing the species abundance signals or the difference in gene copy numbers between strains of *F. prausnitzii*.

## Conclusions

The investigation into function-species relationships undertaken here highlights some important aspects of microbiome studies and the possible inferences that can be made from such information. Although there are potential pitfalls with analysis of abundance of functions within a microbiome as has been done here such as insufficient sampling depth or incomplete sequencing of all DNA fragments, such approaches have revealed marked differences previously [5,17]. It was found that the abundance of components of the peptides/nickel transport system differed between low and high BMI related samples, likely indicating a link between this system and obesity although such a correlation would require validation on other datasets. Taxonomic assignment of KO-associated reads showed that within the peptides/nickel transport system, there are multiple species associated with each KO, with dominance by one species being rare (Table 2). There are numerous possible reasons for this inconsistency of dominant species between KOs. As it is highly implausible that each protein is being created by different species and somehow incorporated separately into the transport systems, it is more likely LGT has resulted in operon or single gene transfers between organisms. This would result in conflicting phylogenetic relationships as observed here and makes determination of the true species involved in pathways difficult. This situation is likely due to the high degree of LGT known to occur in the human gut [18-20]. Although the presence of *F*. *prausnitzii* in all five KO sets may indicate that this species is one of the dominant organisms involved in this pathway, such extrapolation cannot be confirmed without knowing the exact history of LGT events within the microbiome, or with much deeper sequencing that allows for assembly of large genomic fragments as was performed in [21]. Therefore further insight into detecting lateral gene transfer within the microbiome and determining the true species involved in each pathway is required before accurate relationships between species, functions and host properties such as disease be made with confidence.

Analysis of the peptides/nickel transport complex with *F*. *prausnitzii* revealed multiple operons associated with this function, each of which appeared to have been acquired through lateral gene transfer. Previous work on *Fusobacterium nucleatum* found an iron transport complex within the genome that resulted both from LGT of an entire operon and separate LGT events of single genes from multiple strains of other species resulting in two other operons of heterogeneous origins [22]. Within *F*. *prausnitzii* it appears that a similar scenario has occurred within the peptides/nickel transporter with six operons types discovered. It was determined that each operon arose from separate LGT events through analysis of congruent gene trees within the operon (Additional file 4: Figure S3), which is a strong indicator of LGT [22,23]. Five of the six operon types appear to be derived from the transfer of the whole operon into strains of *F*. *prausnitzii*, though the presence of the same operon type in some but not all strains suggests such transfers occurred prior to the divergence of certain strains. The remaining operon which was only found in a complete form within strain A2-165 appears to have been acquired from multiple sources, with the majority of the genes derived from *Lachnospiraceae* bacterium 3_1_57FAA_CT1 with the two ATP-binding related genes derived from other sources (Additional file 4: Figure S3). This may be due to a whole operon transfer followed by subsequent orthologous replacement and demonstrates that although the complexity hypothesis suggests such interactions between a new protein and the pre-existing complex would fail [24], heterogeneous integration can occur and may result in loss of fitness [25,26], if this operon is active. Thus if multiple acquisitions did take place, this could point to a system of gradual gain of novel functions from multiple sources. However, functional assays (such as those performed in [26]) would be required to determine if this operon is transcribed and translated into a complex within this strain.

It may be that all five strains of *F*. *prausnitzii* acquired this transport system from independent sources within their environment (or across habitats from strains of closely related species) via gain-of-function LGT or already possessed the operon which was subsequently overwritten by multiple orthologous replacements, making the history of the lateral gene transfers difficult to trace. The relevance of nickel or short peptide transport within this species is difficult to interpret. Several enzymes such as ureases, hydrogenases, methane reductases and carbon monoxide dehydrogenases use nickel as a cofactor [27] though *F*. *prausnitzii* is not known to have urease activity or many hydrolases [28]. However, a relationship between nickel concentration and butyrate production, a product of *F*. *prausnitzii*[28], has been postulated, and demonstrated in cattle [29]. This could indicate that these strains are influencing the levels of butyrate within the surrounding environment. Concentrations of butyrate and butyrate-producing bacteria have been associated with lower carbohydrate intake [30] and also reduced obesity in mice [31]. This suggests that a subset of the enzymatic functions associated with nickel [27], specifically links to butyrate production and may be connected to levels of obesity with the host, possibly through influence of butyrate production. Additionally, as this transport system can also be involved in more general transport of peptide from two to five amino acid residues in length it could be another unknown function being utilised by this species within the human digestive tract habitat. This module was characterised based upon the Opp complex in *Salmonella typhimurium*[32], which has been shown to be involved in modulating expression of surface-exposed proteins [14]. These proteins may be involved in functions such as sporulation and virulence, both of which have been shown to be important in the human gut microbiome [19,33]. Thus it is possible that this transporter is not involved in nickel regulation but actually modulating the cell surface responses to the digestive tract environment. As it has been shown that low levels of *F*. *prausnitzii* are associated with Crohn’s disease [34] and we have shown here that *F*. *prausnitzii* may also be associated with obesity, it is likely that LGT of systems such as peptides/nickel transport may contribute to host adaptation of this species, as has been observed with LGT in other species [35,36], or play a role in determining the importance of the species within the microbiome. However, further experimental analysis would be required to confirm the link between this membrane transport system and host obesity and also determine is precise function.

Understanding the effect of habitat-directed LGT is a difficult problem. Microbiome data can be utilised to address this as has been shown here. We have found that although an overall signal for clustering of gut-associated organisms was not observed, this is not indicative of a lack of LGT. Each protein tree did not correlate exactly with a species tree as would be usually derived from single-gene studies based on 16S or other marker genes. Subsequent analysis revealed that some species that were clustered together in the protein trees were from taxonomically distant groups (Additional file 4: Figure S3). These species were usually found to be occupying similar environmental niches and were possibly associated with influencing the habitat, in this case the BMI of the host. Thus these findings signify that subsets of species may share genetic information within the environment and such LGT may impact how the habitat as a whole is shaped.

## Methods

### Dataset selection

The dataset of [4] derived from 124 European individuals using Illumina sequencing was used for this analysis. Deep sequencing of samples from these individuals resulted in an average of 4.5 Gb of data per patient, which was further assembled into contigs as described in reference [4]. Associated with these sequences is a range of metadata including BMI, an indicator of the level of obesity of the patient. Low BMI (18 to 22) indicates underweight/healthy patients and a BMI of 30 and above indicates an obese individual. Only lean (low BMI; 34 samples) and obese (high BMI; 33 samples) patients were selected for further analysis to maximise any differences in the microbiome that may be associated with weight.

### Functional assignment of proteins and estimation of abundances within the microbiome metabolic profile

Assembled contigs from each patient were used as input into Orphelia [37] for prediction of open reading frames (ORFs). Any predicted ORFs of length < 150 nucleotides were removed to ensure greater coverage for prediction of function. Prediction of protein function for each ORF was undertaken using UBLAST as implemented in USEARCH version 4.0.38 [38] against a protein dataset derived from 3,181 completed and draft reference genomes obtained from IMG on 4th September 2012. An expectation value cut-off of 10^-30^ was utilised to ensure a high confidence level for the assigned functions. Metabolic functions were linked to a sample’s protein sequence fragments using the KEGG database (v58) [39] with annotations as listed in the IMG database for each genome [14]. If the top hit for an ORF within the reference genome dataset had an associated KEGG Orthologous (KO) group that KO was assigned to the ORF.

A count of each KO within each of the 67 samples was compiled and input to STAMP version 2 [40] in order to detect significant differences in abundances between lean and obese patients, including those that are absent in one but present in the other. Each sample was compared between these two groups using the Welch two-sided *t*-test with Bonferroni multiple test correction. A cut-off p-value of 0.01 was used to identify KOs whose mean abundance differed significantly between low and high BMI samples.

### Phylogenetic reconstruction and taxonomic assignment

Sequences assigned to the same KO set were aligned using ClustalOmega [41] and then trimmed using BMGE [42] with an entropy score of 0.7 and a BLOSUM30 matrix. A hidden Markov model was built from this alignment and all metagenome ORF sequences that were assigned a particular KO were aligned to the reference alignment for that KO using hmmalign. Phylogenetic trees were built for each reference KO alignment using FastTree 2.1 with the JTT substitution model and a gamma distribution [43]. In order to calculate bootstrap support, 100 resampled alignments were built per KO using SEQBOOT of the phylip package [44]. FastTree was then used to create a tree per resampled alignment and the original tree was subsequently compared to these 100 resampled trees to infer bootstrap support per node. Subtrees containing only gut-associated species (as listed in the IMG database [15]) were created and tested for consistency with taxonomy using Chameleon, a visualisation and analysis environment for phylogenetic diversity currently in development.

Classification of metagenomic fragments was undertaken using the Pplacer package v1.1 alpha11 [16]. The taxonomic assignment of each reference sequence was retrieved from the NCBI taxonomy database using Taxtastic (http://fhcrc.github.com/taxtastic) and a Pplacer reference package was created for each KO of interest. Metagenomic sequence fragments were then placed on the tree using Pplacer. This allowed for assignment of each ORF to a taxonomic attribution with a high level of confidence. These classifications were then retrieved using the guppy classification method of Pplacer, which reports the closest taxonomic attribution for each phylogenetically placed read. Differences in abundances of species between lean and obese patients were examined using STAMP version 2 employing the Welch two-sided *t*-test with Bonferroni multiple test correction and a 0.05 p-value cut-off.

## Competing interests

The authors declare that they have no competing interests.

## Authors’ contributions

CJM carried out the study design, analysis, and manuscript preparation and editing. RGB contributed to study design, and manuscript preparation and editing. Both authors read and approved the final manuscript.

## Supplementary Material

Additional file 1 **Figure S1.** Phylogenetic trees of K02031-K02035 (A-E respectively) showing the spread of gut-associated species. Phylogenetic analysis of each set of sequences from proteins within the peptides/nickel transporter showing the spread of gut-associated species (red terminal branches) throughout each tree. Click here for file

Additional file 2 **Table S1.** Consistency index between KO trees of gut-associated species and taxonomic ranks. Subtrees for each KO comprising only gut-associated species were examined for consistency between taxonomy and phylogenetic placement. Click here for file

Additional file 3 **Figure S2.** Phylogenetic tree of gut-associated species for K02031. Phylogenetic analysis of only gut-associated species showing the spread of *Faecalibacterium prausnitzii* (green) and *Clostridium difficile* (red) strains. Click here for file

Additional file 4 **Figure S3.** Phylogenetic analysis of proteins associated with K02031-K02035 within *Faecalibacterium prausnitzii. Protein sequences annotated as being part of the nickel/peptides transporter complex (K02031-K02035) within the five strains of F*. *prausnitzii* were found to fall into one of six subtrees within each protein tree. Each subtree corresponds to an operon as listed in Figure 2. IMG gene object ID locus names for sequences are listed beside the strain name. Branch labels correspond to bootstrap values. Branch lengths are not to scale. (PDF 226 kb) Click here for file
