# Supplementary figures and images for: Lateral gene transfer of an ABC transporter complex between major constituents of the human gut microbiome
Source: BMC Microbiol. 2012 Nov 1;12:248. doi: 10.1186/1471-2180-12-248 (PMC3534369; doi:10.1186/1471-2180-12-248)

A)

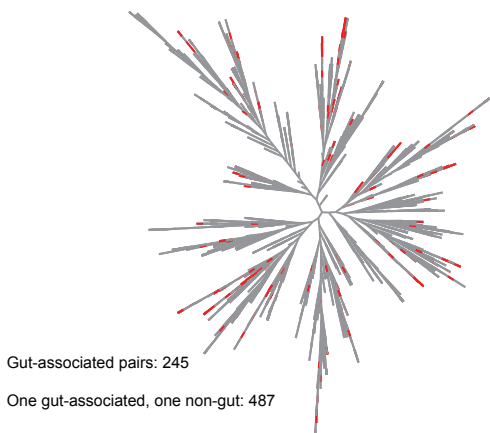

B)

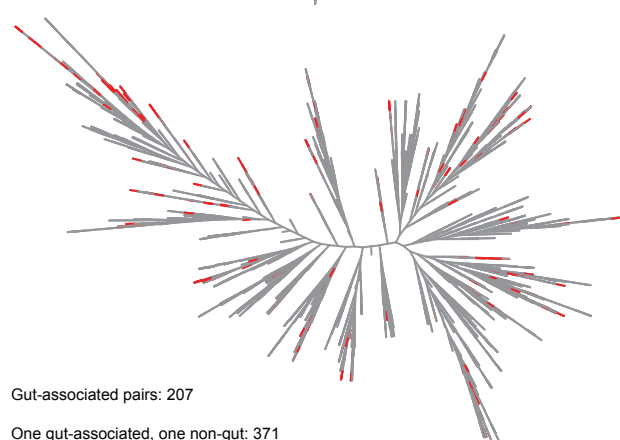

C)

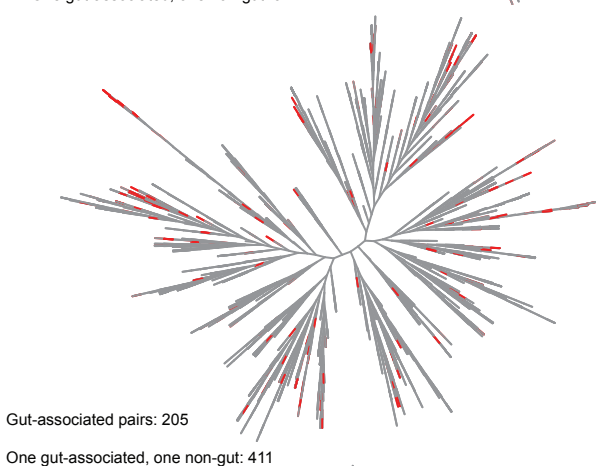

D)

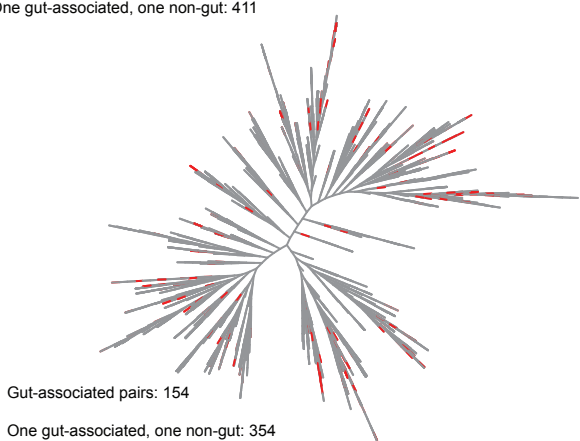

E)

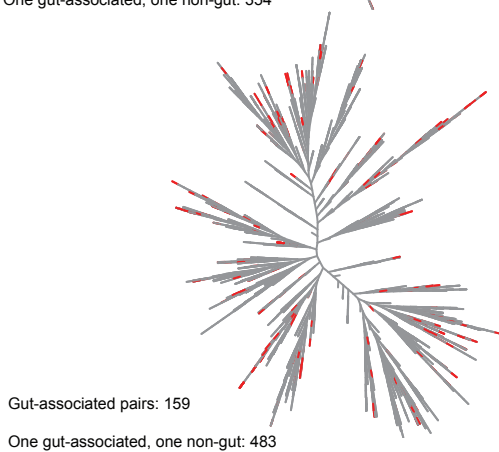

Supplement: Additional file 1 — Figure S1. Phylogenetic trees of K02031-K02035 (A-E respectively) showing the spread of gut-associated species. Phylogenetic analysis of each set of sequences from proteins within the peptides/nickel transporter showing the spread of gut-associated species (red terminal branches) throughout each tree. [file 1471-2180-12-248-S1.pdf]

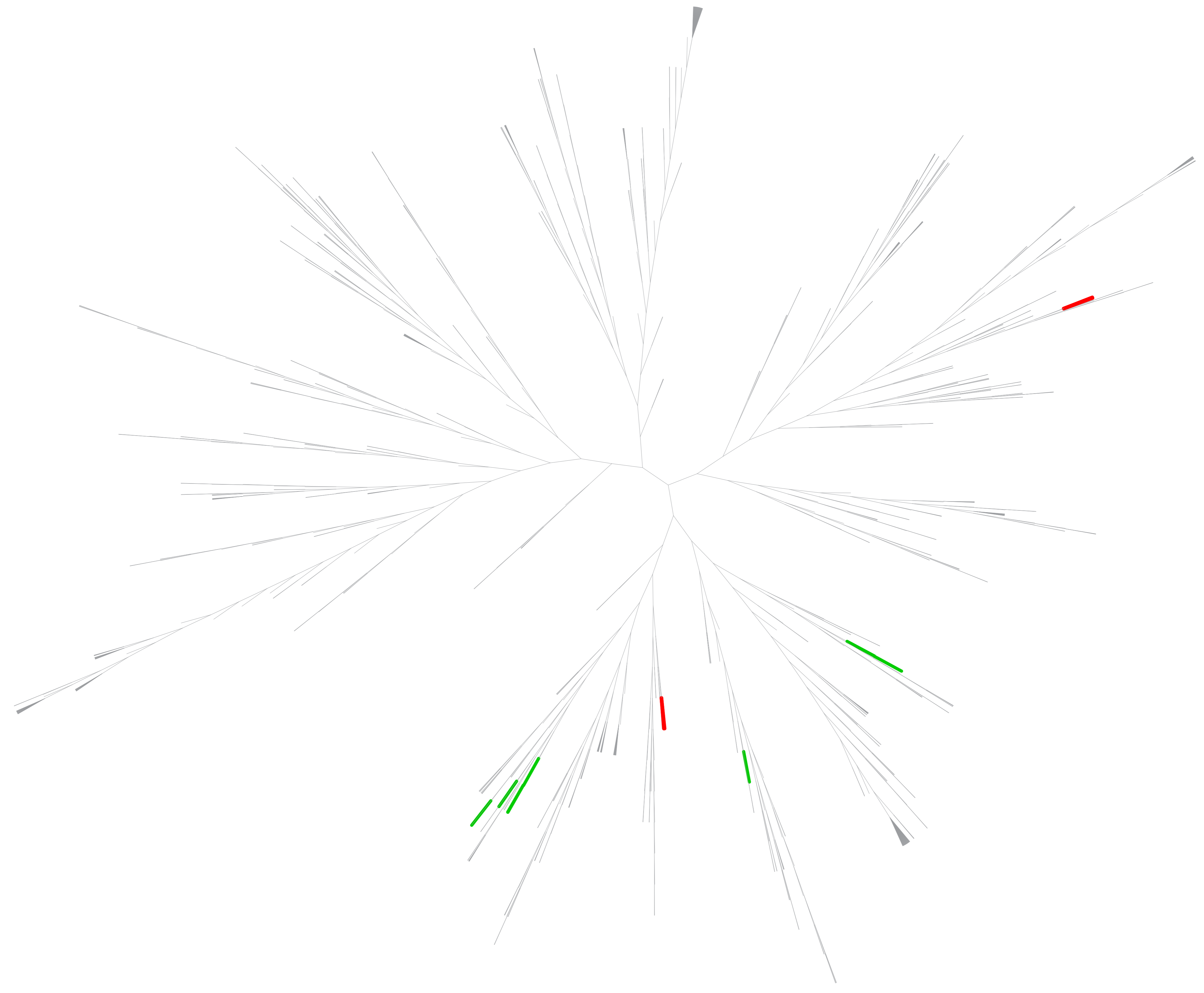

Supplement: Additional file 3 — Figure S2. Phylogenetic tree of gut-associated species for K02031. Phylogenetic analysis of only gut-associated species showing the spread of Faecalibacterium prausnitzii (green) and Clostridium difficile (red) strains. [file 1471-2180-12-248-S3.pdf]

Operon 1

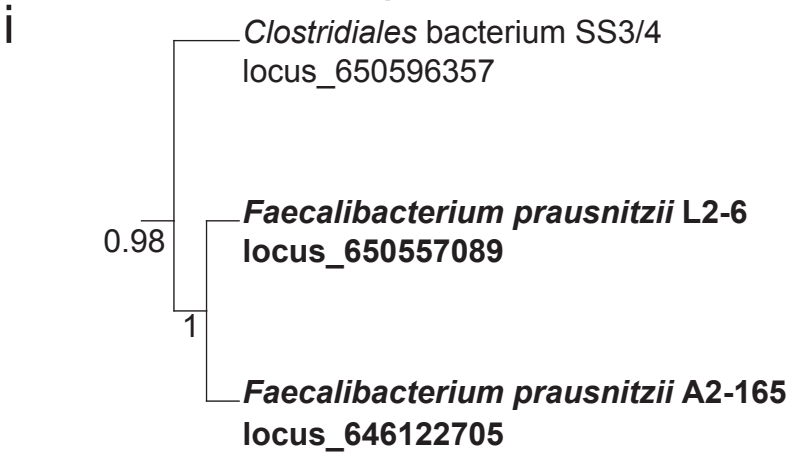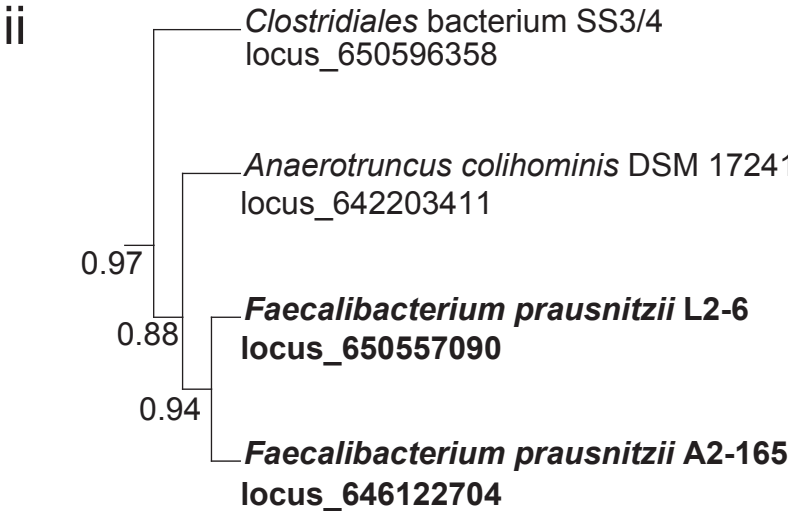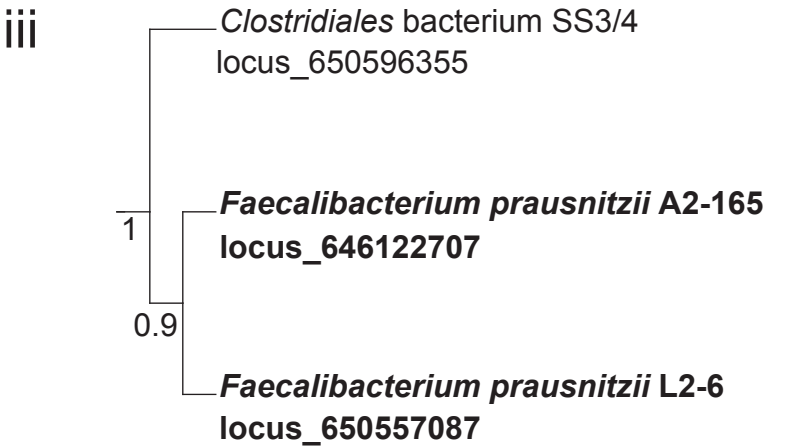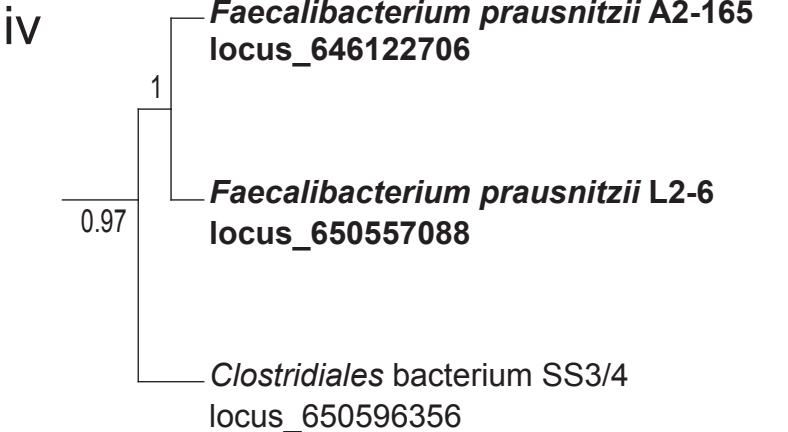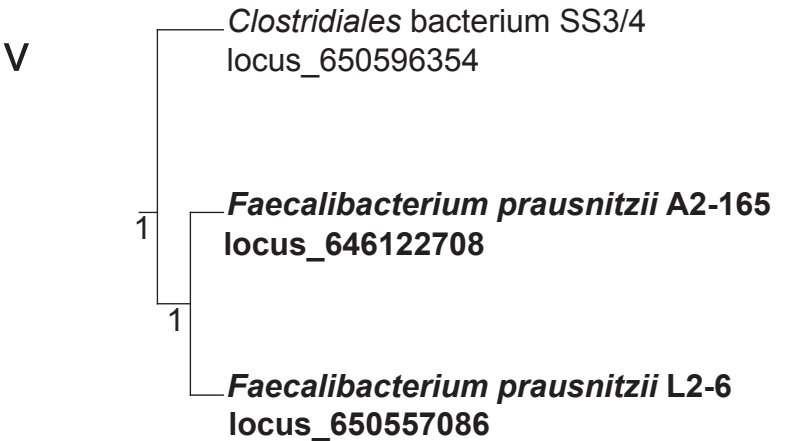

Operon 2

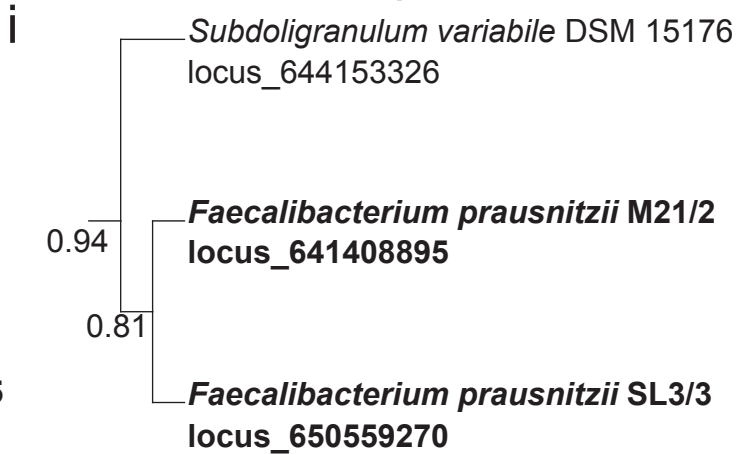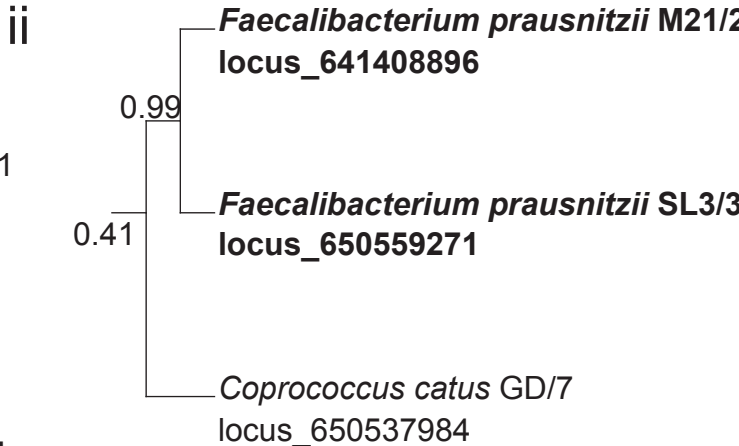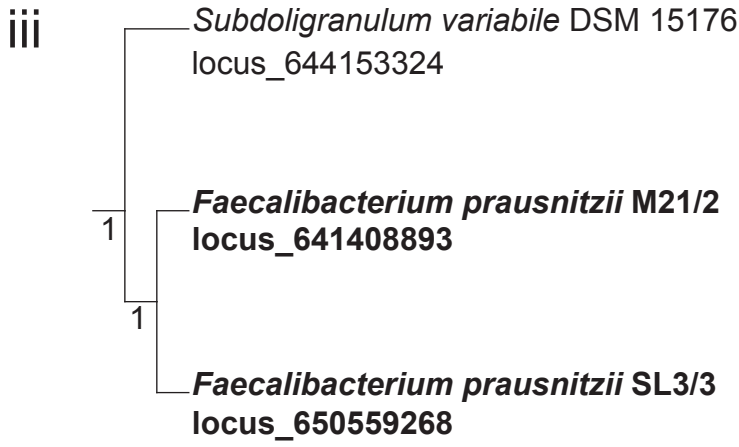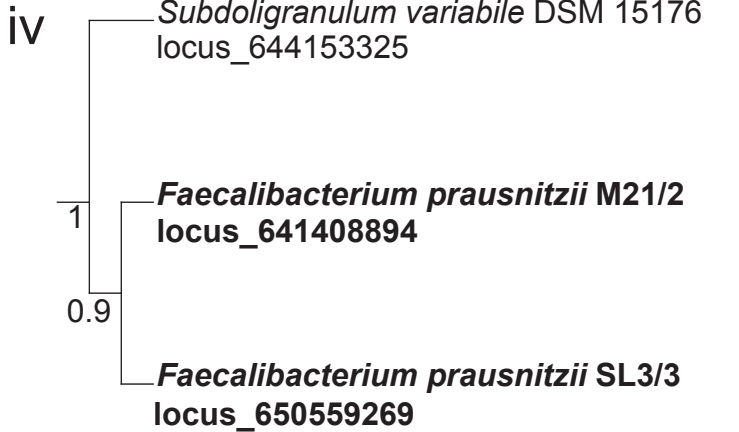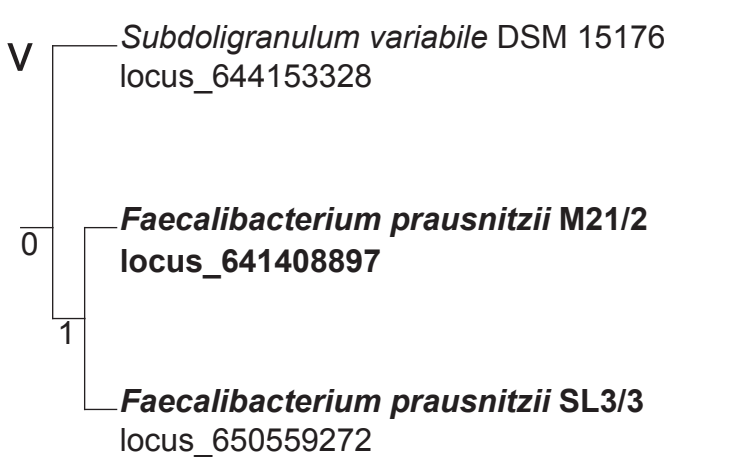

Operon 3

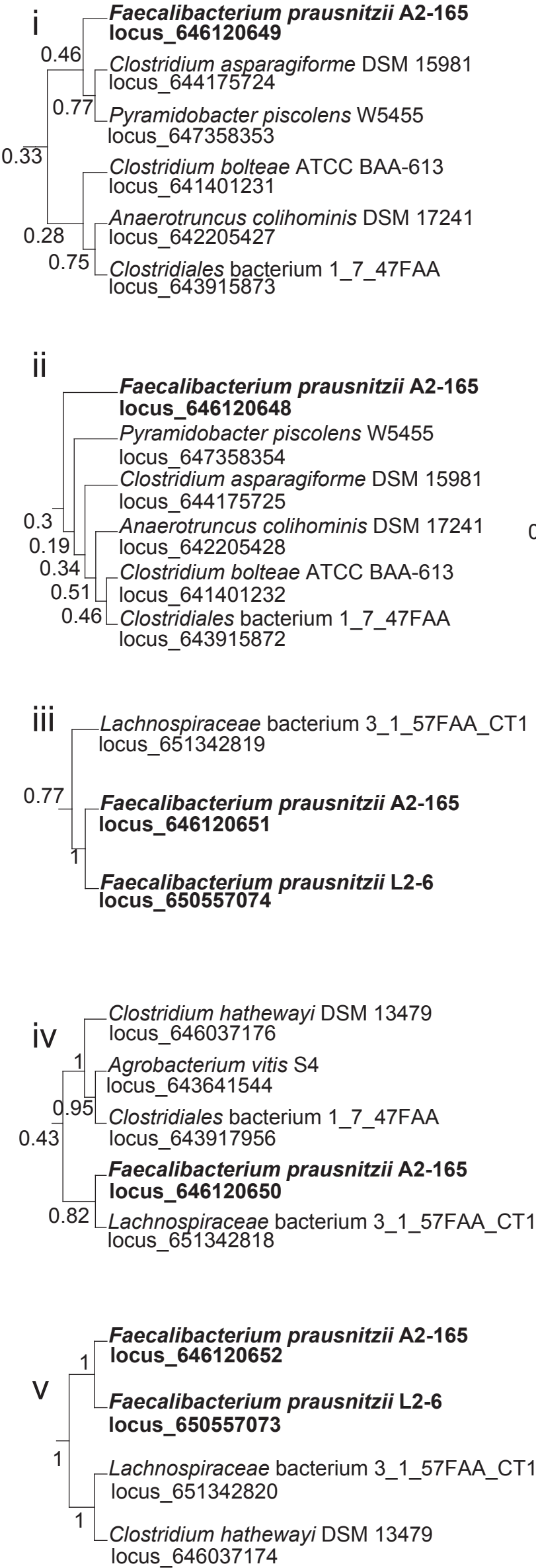

Operon 4

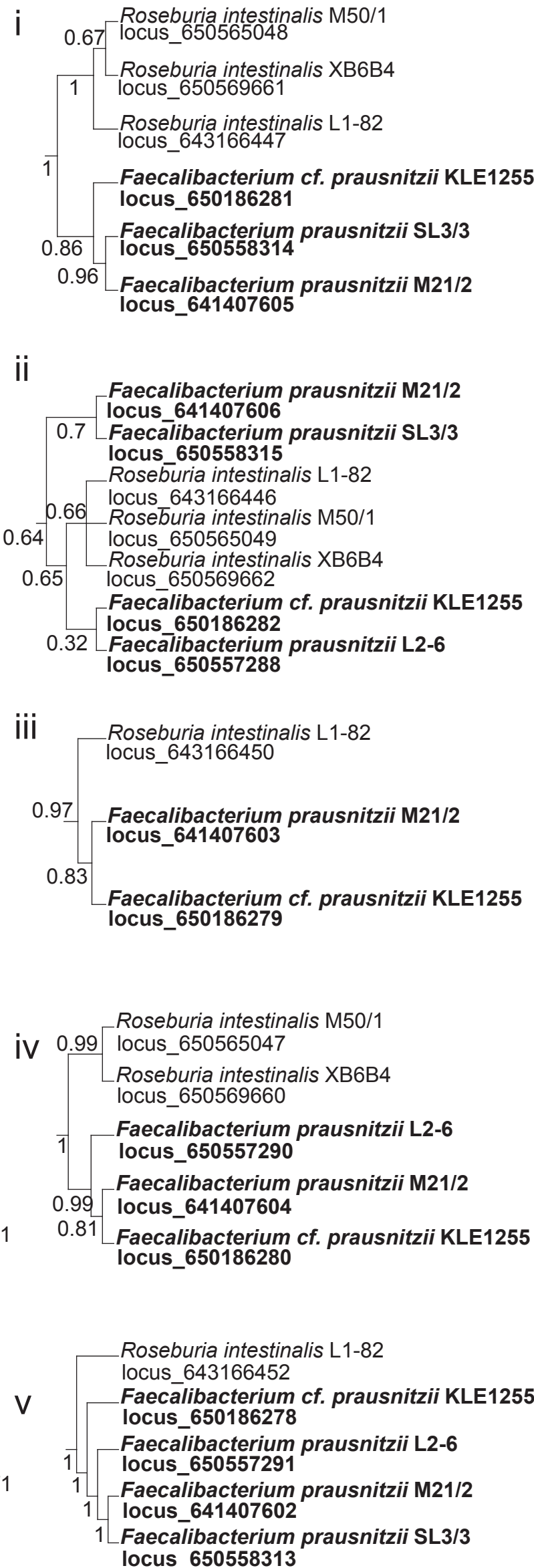

Operon 5

i

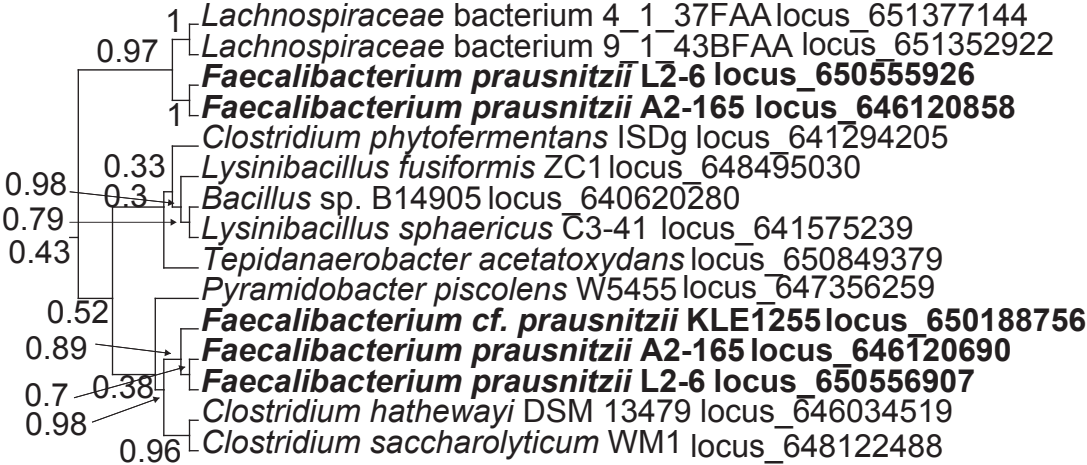

ii

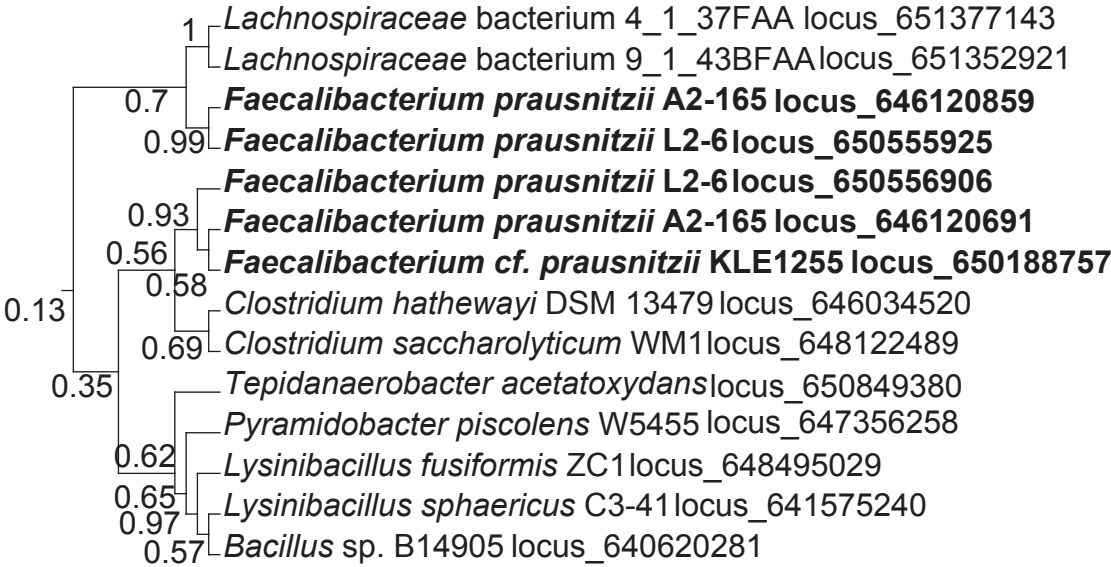

iii

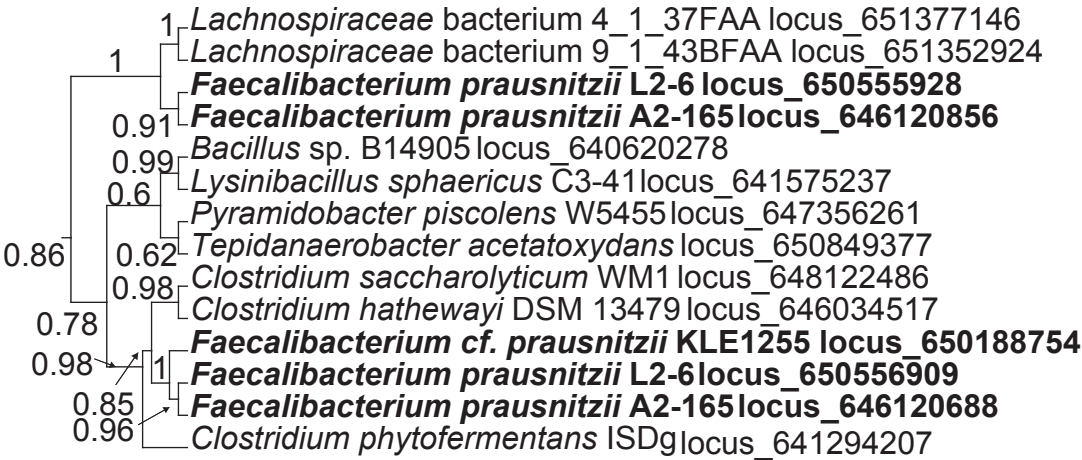

iv

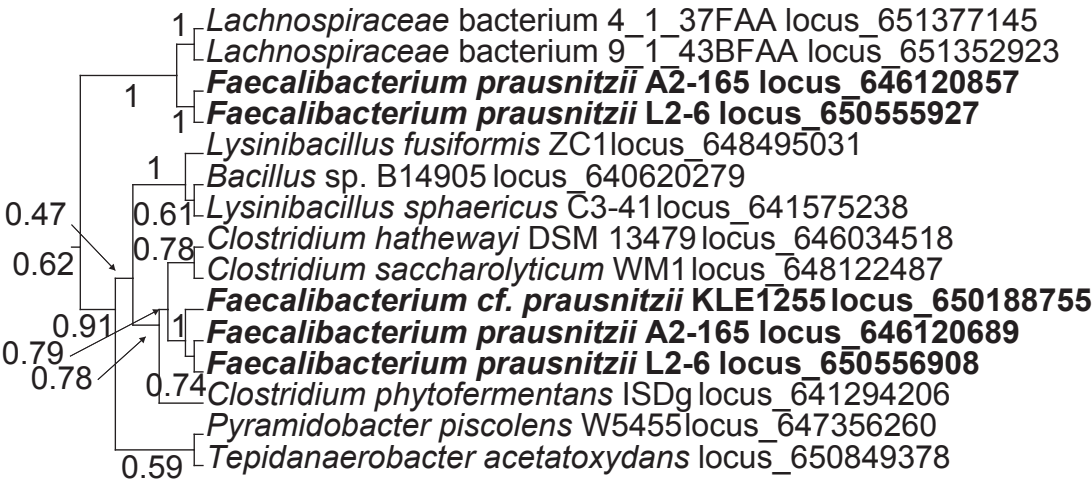

v

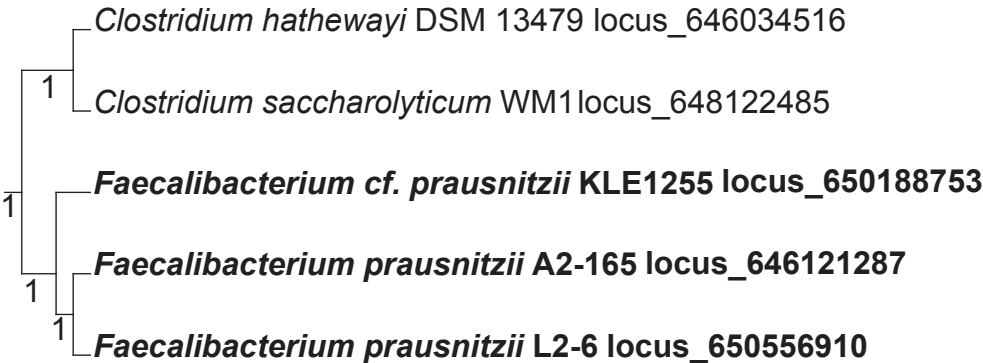

Operon 6

i

ii

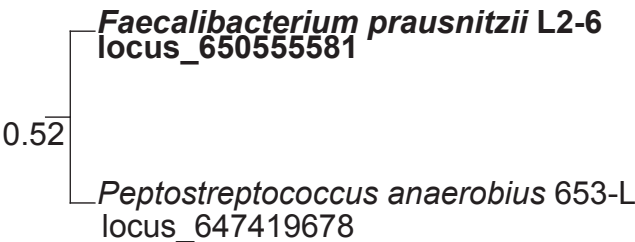

iii

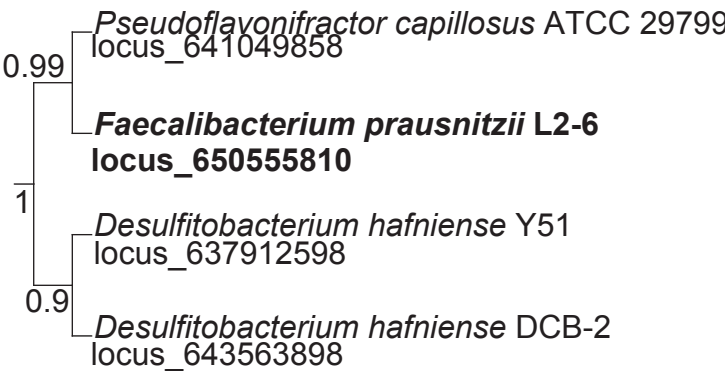

iv

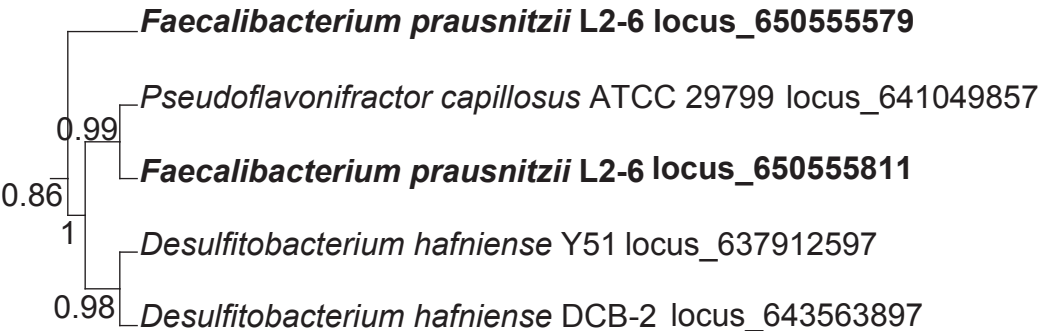

v

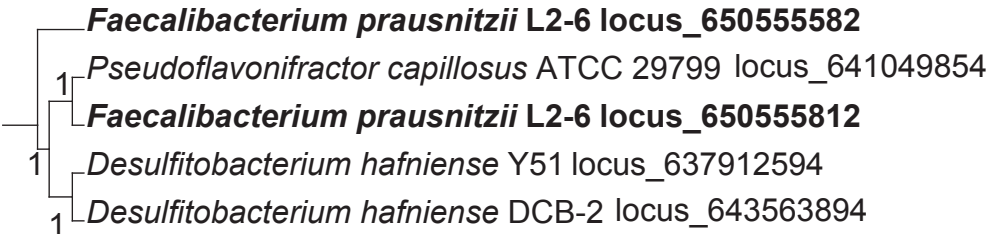

Supplement: Additional file 4 — Figure S3. Phylogenetic analysis of proteins associated with K02031-K02035 within Faecalibacterium prausnitzii. Protein sequences annotated as being part of the nickel/peptides transporter complex (K02031-K02035) within the five strains of F. prausnitzii were found to fall into one of six subtrees within each protein tree. Each subtree corresponds to an operon as listed in Figure 2. IMG gene object ID locus names for sequences are listed beside the strain name. Branch labels correspond to bootstrap values. Branch lengths are not to scale. (PDF 226 kb) [file 1471-2180-12-248-S4.pdf]
