# Supplementary material for: Lateral gene transfer of an ABC transporter complex between major constituents of the human gut microbiome
Source: BMC Microbiol. 2012 Nov 1;12:248. doi: 10.1186/1471-2180-12-248 (PMC3534369; doi:10.1186/1471-2180-12-248)
Supplement: Additional file 2 — Table S1. Consistency index between KO trees of gut-associated species and taxonomic ranks. Subtrees for each KO comprising only gut-associated species were examined for consistency between taxonomy and phylogenetic placement. [file 1471-2180-12-248-S2.pdf]

	K02031	K02032	K02033	K02034	K02035
**Species**	0.96	0.96	0.95	0.94	0.93
**Genus**	0.62	0.64	0.64	0.62	0.6
**Family**	0.44	0.44	0.45	0.45	0.39
**Order**	0.37	0.39	0.39	0.39	0.32
**Class**	0.25	0.26	0.23	0.26	0.19
**Phylum**	0.34	0.32	0.29	0.34	0.2
